# Advanced Parkinson’s Disease Treatment Simplification and Long-Term Outcomes with Levodopa Carbidopa Intestinal Gel: COSMOS Romanian Subanalysis

**DOI:** 10.3390/brainsci11121566

**Published:** 2021-11-27

**Authors:** Mihaela Adriana Simu, Dragoș Cătălin Jianu, Adriana Octaviana Dulamea, Viorelia Adelina Constantin, Diana Popescu, Juan Carlos Parra, József Attila Szász

**Affiliations:** 1 Department of Neurology, “Victor Babeș University of Medicine and Pharmacy, 300041 Timișoara, Romania; mihaelasimu6713@gmail.com; 2 Neurology Clinic, Fundeni Clinical Institute, “Carol Davila” University of Medicine and Pharmacy, 020021 Bucharest, Romania; 3 Department of Neurology, Emergency Clinical County Hospital Târgu Mureș, 540136 Târgu-Mureș, Romania; vioreliaconstantin@yahoo.com; 4 AbbVie Society with Limited Responsibilities, 020276 Bucharest, Romania; diana.popescu@abbvie.com; 5 AbbVie Incorporated, North Chicago, IL 60085, USA; juancarlos.parrariaza@abbvie.com; 6 Department of Neurology, “George Emil Palade” University of Medicine, Pharmacy, Science and Technology, 540139 Târgu-Mureș, Romania; szaszneuro@yahoo.com

**Keywords:** Parkinson’s disease, levodopa carbidopa intestinal gel, LCIG, monotherapy, device aided therapies, DAT, COSMOS, routine clinical practice

## Abstract

The aim of the COmedication Study assessing Mono- and cOmbination therapy with levodopa-carbidopa inteStinal gel (COSMOS) was to assess the use of levodopa/carbidopa intestinal gel (LCIG) as monotherapy in patients with advanced Parkinson’s disease (APD) in routine clinical practice. COSMOS was an international observational study with one cross-sectional visit and retrospective data collection. In Romania, 95 adult patients with APD on LCIG treatment for at least 12 months were enrolled and stratified according to their LCIG therapy after 12 months: monotherapy (without any add-on PD medication), monotherapy with night PD medication and LCIG + add-on medication. Compared to the moment of LCIG initiation, the percentage of patients on monotherapy increased at three months after LCIG initiation and remained constant up to 12 months, when 30.5% of the patients were on LCIG monotherapy and 11.6% were on monotherapy with night medication. “Off” time and “On” time with dyskinesia decreased from LCIG initiation to patient visit in all groups. LCIG monotherapy with or without night medication may provide a simplified treatment option for selected APD patients, with long-term efficacy similar to that of LCIG plus add-on medication.

## 1. Introduction

Levodopa, a precursor of dopamine, is currently considered the most efficient therapy for Parkinson’s disease (PD) [1]. However, long-term use of levodopa is associated with the development of motor fluctuations with alternating periods of good and poor symptom control and dyskinesia [2]. Possible causes of these motor fluctuations include progressive degeneration of the dopaminergic neurons involved in the conversion of levodopa to dopamine, pulsatile stimulation of receptors after oral levodopa administration [3,4,5], and gastrointestinal dysfunction with variable gastric emptying rates, as well as impaired intestinal absorption, a symptom developed in many patients over the course of the disease [6]. Thus, as PD progresses, oral levodopa administration may become inefficient in symptom control [3,6]; therefore, treatment strategies based on continuous drug delivery have been developed.

Levodopa/carbidopa intestinal gel (LCIG) is continuously delivered to the upper intestine, ensuring more stable levodopa plasma levels than oral levodopa therapy, reducing motor fluctuations, and improving some non-motor symptoms commonly associated with chronic oral levodopa treatment [7,8,9]. Clinical trials and post-marketing observational studies on the use of LCIG in routine care conditions have demonstrated LCIG efficacy and safety [10,11,12,13,14,15] and long-term improvements in motor symptoms and quality of life in patients with advanced PD (APD) [7,16,17,18].

In parallel with the disease progression, several combinations of different dopaminergic therapies are usually added to control APD motor symptoms, in addition to medication for non-motor symptoms and comorbidities, thus, leading to polypharmacy with complex dosing schedule and a negative impact on patients’ adherence [19,20]. A simplified treatment regimen in terms of dosing or formulation, supportive care, and counseling are interventions that have shown to successfully improve patients’ adherence and APD control [19]. LCIG monotherapy provides continuous dopaminergic stimulation, reduces the PD-related pill burden, avoids negative effects of oral comedications on adherence, and reduces potential drug-to-drug interactions [21,22,23,24]. Data from clinical trials and post hoc analyses of observational studies showed that monotherapy or a significant reduction of add-on medication may be achieved after LCIG initiation [7,21,22,23,24,25]. Available literature suggests that LCIG monotherapy may achieve effective control of both motor and non-motor symptoms in patients with APD, leading to improved quality of life [25]. However, limited data is available from routine clinical practice on the usage of LCIG as monotherapy or in combination with other PD medication, as well as on the management of add-on medication during LCIG initiation and during long-term therapy [25].

COmedication Study assessing Mono- and cOmbination therapy with levodopa-carbidopa inteStinal gel (COSMOS) was a multinational study designed with the aim to assess the usability of LCIG as a monotherapy or in combination with add-on PD medications in patients with APD in routine clinical practice [26]. Here, we present the results for the Romanian patients enrolled in this study.

## 2. Materials and Methods

### 2.1. Study Design

COSMOS was an international observational study with a retrospective and cross-sectional design, conducted between 2nd of January 2018 and 31st of January 2019 in 14 countries (clinicaltrials.gov NCT03362879). The study consisted of one cross-sectional visit and observational retrospective data collection from the time prior to LCIG initiation and during LCIG treatment (at 3, 6, 9, and 12 months from initiation) [26]. In Romania, patients were enrolled in four study centers, hospitals with experience in treating APD patients and using LCIG therapy. All participants were informed about the study and provided a written informed consent before study inclusion. The study was conducted in accordance with the Declaration of Helsinki, and in Romania, the study-related documents were approved by the National Bioethics Committee of Medicines and Medical Devices with the approval number 13SNI/11.09.2017.

### 2.2. Study Objectives

The primary endpoint of the study was to assess the percentages of APD patients on LCIG monotherapy immediately after LCIG initiation (after permanent system placement) and at 3, 6, 9, and 12 months after LCIG initiation [26]. The secondary endpoints of this study were to describe demographic and clinical characteristics of APD patients eligible for LCIG, PD medication management and the main reasons justifying its use at LCIG initiation and during long-term LCIG treatment, LCIG dosage dynamics over time and reasons for substantial dose adjustments, as well as clinical outcomes in treatment groups [26].

### 2.3. LCIG Monotherapy and Group Stratification

LCIG monotherapy was defined as the use of LCIG infusion with no other add-on PD medication. Patients were stratified according to their LCIG regimen at 12 months, as follows: patients on monotherapy (including patients without any add-on PD medication at 12 months after LCIG initiation), patients with monotherapy with night medication (including patients receiving add-on PD therapy only in the evening after the daily LCIG therapy infusion hours were completed), and polytherapy (including patients on LCIG plus any add-on medication for PD) [26]. For the Romanian sub-analysis, we further stratified the polytherapy group in LCIG plus add-on PD therapy used during the day and LCIG plus day and night add-on PD therapy. In addition, patients were grouped according to their LCIG treatment duration from initiation to patient visit. No patient in the COSMOS study was prospectively followed-up after the study visit, therefore, we have treatment-related information only up to the study visit.

### 2.4. Study Population

Inclusion criteria. Consecutive APD adult patients on LCIG treatment for at least 12 months were included, who received continuous LCIG treatment for at least 80% of the time in the previous year before study inclusion. Additionally, each participant had to be treated by the same investigating physician who initiated and monitored LCIG treatment up to the study visit.

Exclusion criteria. Participants were excluded if they were participating in another clinical study during the LCIG therapy or if they had limited language skills and motivation that would have precluded the completion of study-related questionnaires.

### 2.5. Data Collection

As part of the study procedures, data related to study centers, physicians involved, and patients’ data were collected. The information on study centers was related to the type of institution, the average number of PD and APD patients seen per year, the frequency of routine visits for APD patients on device aided therapy (DAT), and the number of specialist physicians working with PD patients. To describe the participating physician profile, the following data were collected: therapeutic specialty, the average number of PD, APD, and APD patients managed with DAT per year, years of LCIG therapy experience, and the use of standard treatment algorithms and established guidelines for PD treatment.

Regarding eligible patients, data on demographics (age, gender, occupation, education), PD history (duration, age at diagnosis, time from PD diagnosis to onset of motor fluctuation, morning akinesia, wearing off, dyskinesia, and LCIG initiation), clinical status immediately prior to/at LCIG initiation, and comorbidities were collected at patient visit or from the medical records. LCIG infusion dose details were collected in relation to the study visit and all patient visits scheduled according to the standard of care. Patient-reported outcomes collected at study visit were the quality of life (assessed by 8-item Parkinson’s Disease Questionnaire [PDQ-8]) [27], symptoms of impulsive compulsive disorder (Questionnaire for Impulsive-Compulsive Disorders in Parkinson’s Disease—Rating Scale [QUIP-RS]) [28], nocturnal disabilities and sleep disorders (Parkinson’s Disease Sleep Scale-2 [PDSS-2]) [29], and patients’ beliefs, attitudes, and concerns regarding their medication (Beliefs Medication Questionnaire [BMQ]) [30]. Unified Parkinson’s Disease Rating Scale (UPDRS) [31] was used to assess mentation, behavior, and mood (UPDRS I), daily living activities (UPDRS II), motor symptoms (UPDRS III), complications of therapy (UPDRS IV), and modified Hoehn and Yahr Stage score (UPDRS V) before LCIG initiation and at the study visit. Non-motor symptoms were assessed using the NMS Scale (NMSS) [32] and cognitive impairment by Mini-Mental State Examination (MMSE) [33].

Adverse events, including events considered as possibly or probably related to LCIG therapy (adverse drug reactions, ADR), pregnancies, and all product complaints were reported at the study visit and retrospectively for the time interval between LCIG therapy initiation and study visit.

### 2.6. Statistical Analysis

Statistical analyses were performed in SAS^®^ package, version 9.4 (SAS Institute Inc., Cary, NC, USA) and R 4.0.2 language. Patients’ data and secondary outcomes for patients with available results were analyzed separately for each therapy group. Missing data were not imputed and patients with missing data were not included in group analyses. Quantitative data were described by the mean and standard deviation (SD) and qualitative data by frequency distribution. Non-parametric Wilcoxon or Mann–Whitney–Wilcoxon tests were employed, along with Student’s *t* tests, even for interval variables, to detect differences in distributions. Cohen’s d and Cliff’s delta effect sizes were reported. Wilcoxon’s signed rank tests were used to compare before-after data. Pearson’s correlation coefficient between interval data was calculated.

## 3. Results

### 3.1. Study Center and Physician Characteristics

In Romania, four university hospitals with expertise in PD and treating on average 160.0 ± 114.3 PD and APD patients/year (ranging between 40 and 300 patients) were included as study centers. The mean frequency of routine visits in these study centers for APD patients on DAT was 4.0 ± 0.8/year.

Eight neurologists were involved as study investigators from these study centers. The mean number of PD and APD patients treated per year by these investigators was 120.0 ± 96.8 and 47.1 ± 33.4, respectively. These physicians treated with LCIG a median of 15 patients per year (range: 10–70). The mean number of years of experience with this therapy was 6.4 ± 1.7 years. In terms of PD therapy, seven (87.5%) physicians reported using international PD guidelines and recommendations and four (50.0%) reported using national guidelines and recommendations. LCIG monotherapy was preferred by two (28.6%) of the participating physicians.

### 3.2. Description of the Study Population

The study centers in Romania enrolled 95 patients fulfilling the inclusion criteria and without any exclusion criteria. At study visit, almost half of them (48.4%) had been treated with LCIG up to two years, 21.1% up to three years, 12.6% up to four years, 7.4% up to five years, and 10.5% had more than five years of treatment. The study did not include prospective monitoring of enrolled patients after the study visit; therefore, the treatment duration was calculated from LCIG initiation to study visit.

Table 1 presents the characteristics of patients at 12 months after LCIG initiation. According to the study group, on average, patients were diagnosed with PD 9.5 to 11.3 years before LCIG initiation, at a mean age of 56.0 to 57.7 years. Time to LCIG initiation, motor fluctuations onset, wearing off, and dyskinesia were similar in all treatment groups. PD phenotype was almost equally split between tremor-predominant, akinetic-rigid, and mixed forms. More than 80% of the patients had mild cognitive impairment according to MMSE score calculated at patient visit, irrespective of the LCIG treatment duration group. In all study groups, the main reason to start LCIG therapy was the presence of disabling motor fluctuations/Off periods.

#### Conventional Treatment at LCIG Initiation

Information related to oral medications administered at LCIG initiation were available for 47 out of 95 patients (Supplementary Table S1). Dopamine agonists were the agents mentioned most of the time (23 out of 47 patients, 48.9%), followed by equal numbers of combinations including levodopa and rasagiline (19 out of 47, 40.4%).

### 3.3. Frequency of Monotherapy, Time to Monotherapy, and Monotherapy Duration

**Figure 1:**
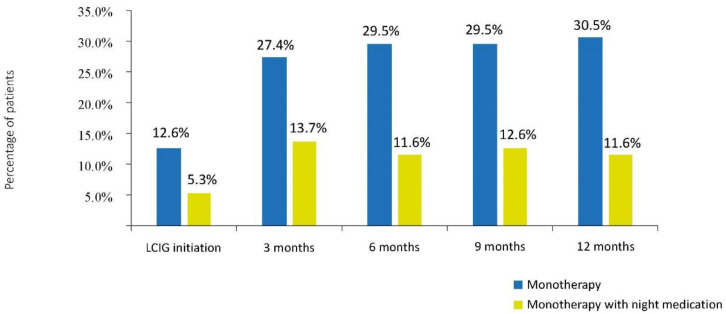
The percentages of patients with advanced Parkinson’s disease on LCIG monotherapy at indicated time points. LCIG = levodopa-carbidopa intestinal gel.

Compared to treatment initiation moment, the LCIG monotherapy use increased from 12.6% to 30.5% at 12 months. The percentage of patients on monotherapy with night medication increased from 5.3% at LCIG initiation to 11.6% at six months and remained relatively constant up to 12 months following LCIG initiation (Figure 1). Mean monotherapy duration from LCIG initiation to patient visit was 770.7 ± 571.2 days in the monotherapy group and 655.7 ± 289.1 days in the monotherapy with night medication group.

**Figure 2:**
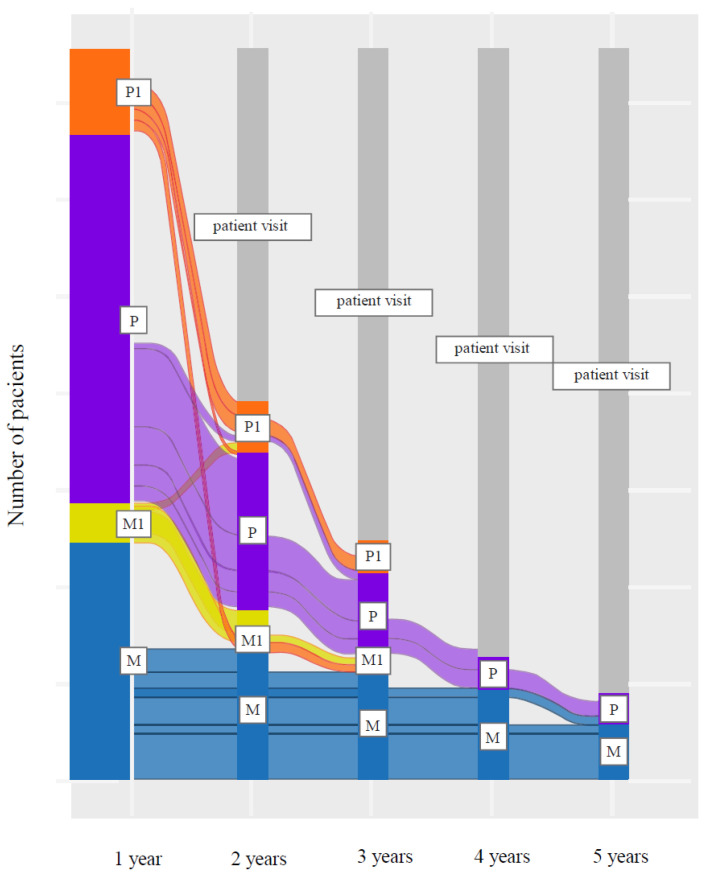
Treatment dynamics after 12 months of LCIG therapy up to patient visit. Note: treatment groups were followed up to patient visit, which may have occurred at different time points for each patient. The treatment duration was calculated from LCIG initiation up to patient visit. The grey vertical bars indicate patient visit moment. The type of treatment after the patient visit is not available, since the study did not include a prospective monitoring after the patient visit. M = LCIG monotherapy; M1 = LCIG + night therapy; P = LCIG + day add-on therapy; P1 = LCIG + day and night add-on therapy. LCIG = levodopa-carbidopa intestinal gel.

Of the 95 patients enrolled, the reference groups considered after 12 months of treatment with LCIG included 31 patients with LCIG monotherapy, five patients with LCIG and night therapy, 48 patients with LCIG and add-on PD therapy used during the day, and 11 patients with LCIG plus day and night medication. To be included in the study, a minimum of 12 months of treatment with LCIG was required. However, at study visit, patients had different treatment durations, since LCIG has been initiated at different moments in time. Figure 2 is based on treatment data recorded in the medical charts (LCIG and any oral PD medication) and presents the treatment dynamics of the Romanian cohort stratified at 12 months.

### 3.4. LCIG Infusion Monotherapy and Add-On Therapy during Follow-Up

**Figure 3:**
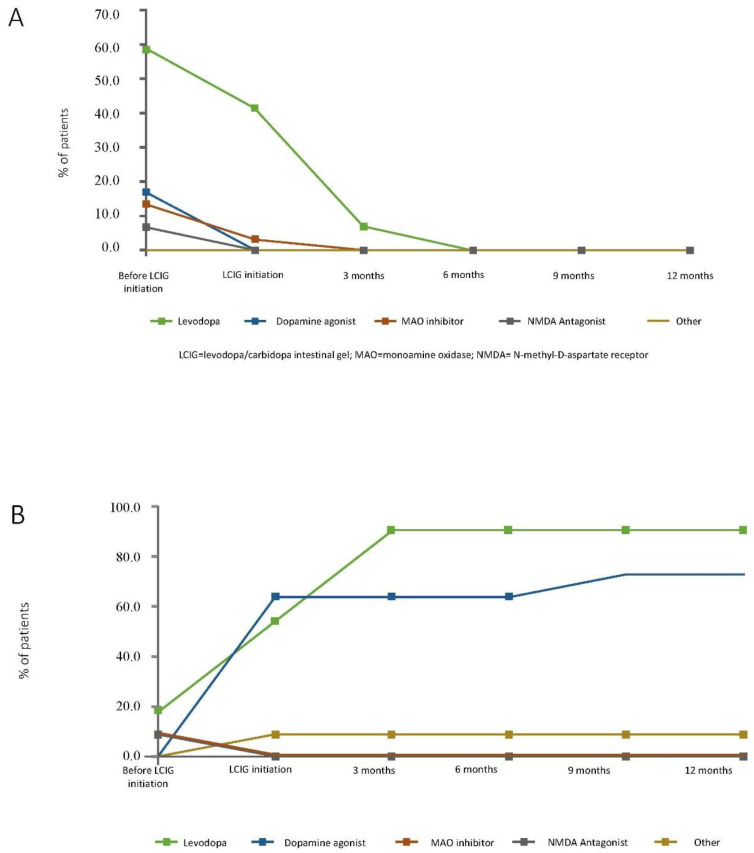

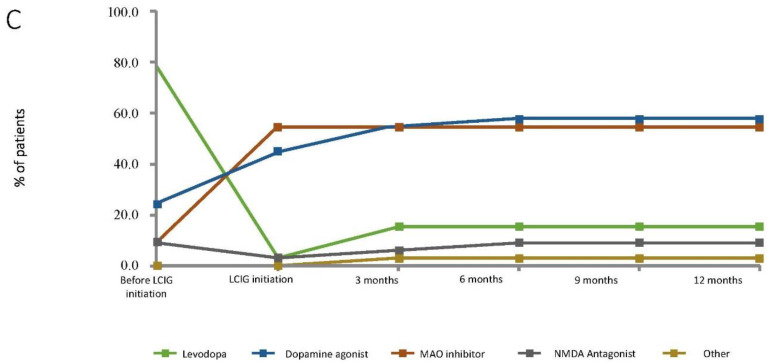
Percentages of patients with add-on therapy over time in the LCIG monotherapy (panel **A**), monotherapy with night medication (panel **B**), and LCIG plus add-on medication (panel **C**) groups. LCIG = levodopa-carbidopa intestinal gel; MAO = monoamine oxidase; NMDA = N-methyl-D-aspartate. Levodopa use included levodopa/carbidopa, levodopa/carbidopa/entacapone, and levodopa/benserazide.

The percentage of patients from the monotherapy group (as it was defined at 12 months of LCIG treatment) using an add-on PD medication at LCIG initiation moment decreased over time, thus becoming monotherapy group; at six months all those patients had no add-on therapy and maintained as such until the end of the first year of LCIG treatment. Therefore, at 12 months of LCIG treatment they were defined as monotherapy group (Figure 3).

The percentage of patients from the monotherapy with night medication group (as it was defined at 12 months of LCIG treatment) using a MAO inhibitor or an NMDA inhibitor decreased over time in the first year after LCIG initiation, while the use of dopamine agonists and levodopa add-on therapy increased at LCIG initiation and at three months, respectively, and remained constant thereafter.

In the polytherapy group, the percentage of patients with levodopa combinations, dopamine agonists and MAO inhibitors increased after LCIG initiation and remained constant thereafter (Figure 3). Regarding COMT inhibitor use, levodopa/carbidopa/entacapone was reported in 32 out of 95 (33.7%) patients prior to LCIG initiation. At the time of LCIG initiation, five patients out of 95 (5.3%) received entacapone as add-on therapy. Just one patient out of 95 (1.1%) continued entacapone add-on therapy after the three-month routine evaluation, up to the patient visit.

In all study groups, the main reason to start LCIG therapy was the presence of disabling motor fluctuations/Off periods. The main reasons for using an add-on medication during the first three months following LCIG initiation were the need to start night medication and the need to improve specific symptoms (Supplementary Table S2).

### 3.5. Patient and Physician-Reported Outcomes

UPDRS total scores, complications of therapy scores and modified Hoehn and Yahr stage were stable after at least 12 months of LCIG therapy in all study groups. Significant results at study visit versus LCIG initiation were noted in the group with LCIG monotherapy at 12 months regarding the UPDRS III score increase (29.7 vs. 15.2, *p* = 0.0117), and in the LCIG + add-on medication group for the improvement of daily activities (13.1 vs. 16.3, *p* = 0.0469) (Supplementary Table S3).

Duration of dyskinesia was assessed by UPDRS Part IV and showed similar improvements at study visit as compared to LCIG initiation in all treatment groups: -1.4 ± 2.1 h in the monotherapy group, −1.3 ± 3.7 h in the monotherapy with night medication group, and −1.4 ± 2.0 h in the LCIG + add-on medication group (*p* > 0.05 for all comparisons between groups). “Off” time also showed similar improvements at study visit as compared to LCIG initiation in the monotherapy and LCIG + add-on medication groups (−4.5 ± 3.4 h in the monotherapy group vs. −3.8 ± 1.1 in the add-on therapy group, *p* = 0.8221). The highest improvement in “Off” time duration was observed in the monotherapy with night medication group (−10.1 ± 3.1 h, *p* = 0.0042, as compared to the monotherapy group) (Supplementary Table S3).

Several baseline disease characteristics, such as duration of PD and number of hours per day in “Off” or “On” with dyskinesia were similar between patients across treatment duration groups (Table 2). The number of hours per day in “Off” or “On” with dyskinesia was higher in groups with night medication at 12 months (Supplementary Table S4).

The mean percentage reduction at 12 months from LCIG initiation in “Off” time and “On” time with dyskinesia was 70.3% and 25.2%, respectively (Table 3), corresponding to an actual change in hours of −4.8 ± 3.3 and −0.6 ± 3.4, respectively.

The reduction observed in the number of hours spent in “Off” time from LCIG initiation to patient visit was significant (*p* < 0.0001), with a large effect size (Cliff’s delta = 0.94). The monotherapy group recorded a significantly higher mean reduction than the rest of the groups taken together (*t* = 2.2, df = 17, *p* = 0.042), with a large effect size (d_Cohen_ = 0.8524). The correlations between the change in the number of hours in “Off” and time with PD, age at LCIG initiation, total duration of LCIG treatment until study visit, and total time duration in “Off” at LCIG initiation were negligeable.

Time with dyskinesia decreased significantly from LCIG initiation to patient visit (*p* = 0.04) in patients with polytherapy, with a small effect size (Cliff’s delta = 0.18). The change was not statistically significant in patients with LCIG in monotherapy, overall (*p* = 0.6).

The correlations between the change in dyskinesia and total disease duration, age at LCIG initiation, total duration of LCIG treatment, and number of hours with dyskinesia at LCIG initiation was negligeable.

NMSS was not available for LCIG initiation in any study group. At study visit, the scores were significantly lower in the monotherapy group as compared to the monotherapy with night medication group (*p* = 0.0417 for the difference between groups) and similar to the NMSS score in the LCIG + add-on medication group (*p* = 0.4787 for the difference between groups) (Supplemental Table S3).

At patient visit, the quality of life (as assessed by PDQ-8), symptoms of impulsive compulsive disorder (as assessed by QUIP-RS), and nocturnal sleep (as assessed by PDSS-2) were similar in all treatment groups (p values for the difference between groups > 0.05 for all questionnaire scores). Moreover, the BMQ score assessing patients’ beliefs, attitudes, and concerns on overuse and harms of the currently prescribed medication were low and no difference was observed between treatment groups (Supplemental Table S5). The PDQ-8 scores did not correlate with disease duration, age, and severity of dyskinesia at LCIG initiation, or with the overall treatment duration.

### 3.6. Safety

During LCIG initiation, one ADR considered as possibly or probably related to the LCIG therapy was reported in one patient. This ADR was polyneuropathy and did not result in an LCIG dose change. During the maintenance therapy, three ADRs were reported in different patients. For patients with polyneuropathy no dose change occurred. The reaction related to embedded device resulted in LCIG therapy discontinuation (Supplemental Table S6).

## 4. Discussion

Clinicians treating PD patients have a high number of therapeutic options available, with proven efficacy, as opposed to other neurodegenerative diseases. In Romania, the treatment strategy in early and intermediate PD is consistent with literature data [34,35,36,37].

In advanced stages, before DAT initiation, the LD doses are at the lower limit of the dose interval specified by other publications; however, add-on strategies are more frequently used [35]. A “paradoxical” simplification of the treatment algorithm would be an ideal approach in APD, considering the decreased adherence, progressive cognitive decline, presence of comorbidities, as well as drug interactions [36].

The complex management of APD patients may become even more difficult in Romania, as some conventional treatment options such as amantadine extended release, levodopa inhalation powder, safinamide and several COMT inhibitors (such as opicapone and tolcapone) are not yet available [38]. In local current clinical practice, DAT eligibility is established in a hospital setting, in university clinics acting as movement disorder centers, with dedicated multidisciplinary teams [36,37,38,39,40]. A detailed scoping review on epidemiology, diagnosis, and clinical aspects of PD provided important insight information related to PD management in Romania [41,42].

According to treatment guidelines and recommendations available at the time of COSMOS study, DAT eligibility assessment in Romania was based on low clinical response or persistence of PD symptoms despite maximally optimized conventional therapy [35,43,44]. In clinical practice, DATs are considered in cases when maximal doses and combinations of oral therapies are not optimally controlling the symptoms or based on inability to further increase submaximal doses due to tolerability issues [43]. Accumulation or worsening of dopaminergic adverse effects of add-on therapies is common in our practice.

Although the triple therapy with levodopa (levodopa/carbidopa/entacapone) is recommended by guidelines before assessment of eligibility for DAT, only one third of patients received entacapone in our study. This situation may reflect the challenges occurring in clinical practice in the quest to achieve the balance between formal recommendations to use maximal doses for treatment optimization and individual tolerability of patients with APD [45]. It persists an ongoing dilemma regarding COMT inhibitor use when choosing between potential improvement in wearing off and the risks of worsening of dyskinesia or occurrence of intolerable gastrointestinal effects.

To our knowledge, this is the most comprehensive analysis of patients with advanced PD receiving LCIG in Romania and also the first country analysis of COSMOS study. The design of the COSMOS study allowed enrollment of APD patients initiated on LCIG in monotherapy or LCIG + add-on medication at various moments in time, therefore, providing a picture of the overall APD management in Romanian clinical practice at the moment of the study period. At study visit, almost one third of all 95 patients enrolled had been receiving LCIG for more than three years. The treatment groups (monotherapy with or without night medication and polytherapy) were established based on treatment received at 12 months of LCIG treatment.

Over the years, in COSMOS patients treated with LCIG four years before the study visit, we have observed that night medication has been added predominantly in patients with more hours spent in “Off” state. This could be a confirmation of the link between disease severity and the acknowledged need for higher dopaminergic stimulation through night therapy, in this patient population with important dopaminergic depletion.

The analysis of real-life data collected in the Romanian cohort from the COSMOS study showed that an important percentage of APD patients can be managed on the long term with LCIG monotherapy (monotherapy or in association with add-on night medication). Overall, the percentage of patients on LCIG monotherapy doubled at 12 months from treatment initiation (with or without night medication), with maintenance of achieved improvement of UPDRS scores, as well as reduction of both “Off” time an “On” time with dyskinesia.

Our results are consistent with those reported in the global COSMOS sample [26] and in the previous post hoc analyses of clinical trials and of an observational clinical study showing that LCIG monotherapy represents both a simplified and an efficient therapeutic option in these patients [21,22,23,24,26].

The streamline of treatment strategy comes in the context of several prerequisites for DAT initiation, including the use of maximum tolerated doses of oral medicines without achieving the optimal control of symptoms [43].

In the global COSMOS sample, enrolling 409 patients with APD, an increase in the percentage of patients on LCIG monotherapy in the first three months after therapy initiation, and stable percentages of monotherapy thereafter were also reported. Therapy with a dopamine agonist, MAO inhibitor, and COMT inhibitor was discontinued within short time period after LCIG initiation. The use of entacapone in combination with levodopa/carbidopa at the initiation of LCIG was in 18.1% of patients in the global study and 5.37% in the Romanian cohort. The low values may reflect the relative difficulty or reluctance of some neurologists to adjust the entacapone dose to a significantly reduced dose of levodopa within continuous delivery. The rationale of adding entacapone to LCIG may be useful in cases requiring the reduction of LCIG dose [46]. In our study, entacapone was discontinued in four patients after three months. On long term, entacapone was maintained as add-on to LCIG in only one patient up to the patient visit, a practical approach if either LCIG provided disease control without the need of entacapone, or if individual tolerability issues led to discontinuation of a COMT inhibitor, or both.

The larger sample of patients included in the global COSMOS study allowed the identification of predictors of monotherapy, such as number of motor symptoms, frequent physician visits, and previous use of dopamine agonist inhibitors [26]. Our study also confirms the results from a recently published post hoc analysis of data from six phase three open-label studies showing similar improvements in the “Off” time, “On” time without dyskinesia, activities of daily living, and motor symptoms in the monotherapy and polytherapy groups, in all studies included in the analysis [24].

COSMOS results are supported by the Global long-term registry on efficacy and safety of LCIG in patients with APD in routine care (GLORIA) registry, which also enrolled a large sample of patients with APD treated with LCIG in real-life settings [25]. Of the 356 patients enrolled, 33.0% were on LCIG monotherapy at baseline and 57% of those on LCIG monotherapy remained as such up to the end of the two-year follow-up. Additionally, 16.0% of those on levodopa monotherapy and 8.0% of those on polytherapy switched to LCIG monotherapy by the end of the follow-up. A sustained reduction of dyskinesia duration by two hours, of the “Off” time by five hours, and improvements in the daily living activities, “On” time with dyskinesia, and motor symptoms were observed in the LCIG monotherapy group [25,47]. Both studies support the hypothesis that LCIG monotherapy is effective in the symptoms control of APD and may be achieved, with the aim to reduce polypharmacy and improve patient adherence to PD therapy [24,25].

In the COSMOS study, the focused analysis by treatment type showed a larger reduction in the “Off” time in patients with LCIG plus night medication. As mentioned earlier, although the COSMOS study included patients with different total treatment durations, an overall reduction in the “Off” time with LCIG was maintained. The magnitude of the “Off” time reduction percentage at patient visit, higher than 74%, is higher in patients with supposed more severe disease (e.g., needing more dopaminergic stimulation, as monotherapy + night medication, or polytherapy during the day or polytherapy + night medication). This observation could be important for clinicians initiating LCIG in different types of patients, with various therapeutic needs.

Disease duration at LCIG initiation did not influence the change in the “Off” time and “On” time with dyskinesia as these patients showed comparable benefits. Although the number of patients included in our sub-analysis was low and the results should be interpreted with caution, we emphasize an important recommendation [48,49] to consider DAT eligibility according to other clinical parameters, including five daily levodopa doses and lack of dyskinesia response to amantadine 100–400 mg/day, than PD duration.

In the current analysis of the COSMOS study, only four ADRs were reported, one in the LCIG initiation phase and three during the maintenance therapy. This low number should be interpreted with caution as ADRs were collected retrospectively and, thus, an under-reporting or lack of recording in medical charts may have occurred.

In this analysis of the COSMOS results, LCIG monotherapy was also associated with improvements in patient-reported outcomes comparable to the ones reported by patients on LCIG with add-on medication. At 12 months following LCIG therapy initiation the quality of life, quality of sleep, and compulsive behavior were similar in the groups with LCIG monotherapy and in the group with LCIG plus add-on medication. Sustained improvements in the quality of life with LCIG monotherapy were previously reported in other clinical studies [23] and real-life settings [24]. Sleep disruption is a major issue in PD patients, occurring in up to 98% of the patients [50,51,52,53] and representing an important part of the non-motor symptoms [54]. Sleep fragmentation and nocturnal akinesia are frequently associated with excessive daytime sleepiness and influence daytime functioning. Significant and sustained improvement of sleep disruption with LCIG therapy was described in the GREENFIELD observational study in which LCIG effect on sleep maintained up to three years in the sample of 115 patients followed up to the study end [23]. It has been shown that the effect of LCIG therapy on PD-associated sleep disruption is due to the improvement of motor symptoms during night (restlessness in arms or legs, urge to move arms and legs), improvement of excessive daytime sleepiness [55], and diminished sleep fragmentation as assessed by polysomnography [56].

A formal statistical comparison between the global and local results was not planned and our intent was to present the cohort characteristics and outcomes following the LCIG treatment in the local current practice. Additional statistics allowed us to observe the characteristics of this country-specific patient population: low entacapone use before and after LCIG initiation, split of polytherapy group by the use of night medication, and illustration of patient flow between treatment groups from LCIG initiation to study visit.

Although it offers important information on LCIG therapy in real-world conditions, the current study has several limitations. First, patients were selected upon convenience and this may have caused a selection bias. Moreover, patient-reported outcomes were assessed during the study visit and these are prone to a certain degree of reporting bias. The retrospective collection of patient data was associated with missing data as some investigators did not use all scales or questionnaires or did not record in patient files all data collected in this study. COSMOS enrolled only APD patients receiving LCIG for at least one year, that were not followed up prospectively. It is likely that these prerequisites to have contributed to the low number of AEs related to LCIG reported in the study.

## 5. Conclusions

COSMOS study showed that LCIG monotherapy with or without night medication may provide a simplified, effective treatment option for selected APD patients, providing an improvement in health-related quality of life. Moreover, reduction in the “Off” time and increase of the “On” time without dyskinesia were similar to the one achieved with LCIG in different combinations. At 12 months following LCIG initiation patients with monotherapy showed non-motor symptoms and patient-reported outcomes comparable to the ones reported by patients on LCIG plus add-on medication. Adverse events findings were in line with the safety profile of LCIG. Thus, LCIG monotherapy may provide for many selected APD patients long-term efficacy and a simplification of the treatment regimen, similar to that of LCIG plus add-on medication.

## Figures and Tables

**Table 1:** Characteristics of patients with known therapy status at 12 months after LCIG initiation by study groups (full analysis set).

	LCIG Monotherapy without Night Medication at 12 Months N = 29	LCIG Monotherapy with Night Medication at 12 Months N = 11	LCIG + Add-on Medication at 12 Months N = 33	*p*-Value
***Before LCIG initiation***				
Men, n (%)	19 (65.5%)	10 (90.9%)	15 (45.5%)	0.1083 * 0.1132 ^#^
Education, n (%)				
Primary school Secondary school Professional education University	8 (27.6%) 14 (48.3%) 3 (10.3%) 4 (13.8%)	0 (0.0%) 4 (36.4%) 5 (45.5%) 2 (18.2%)	13 (39.4%) 16 (48.5%) 2 (6.1%) 2 (6.1%)	0.0401 * 0.5848 ^#^
Primary occupation				
Retired On sick leave Unemployed Working full time	27 (93.1) 0 (0.0) 2 (6.9) 0 (0.0)	10 (90.9) 0 (0.0) 0 (0.0) 1 (9.1)	29 (87.9) 1 (3.0) 3 (9.1) 0 (0.0)	0.1828 * 0.6012 ^#^
Age at PD diagnosis, years	56.7 ± 10.0	57.7 ± 10.4	56.0 ± 8.4	0.8921 * 0.5004 ^#^
Time from PD diagnosis to motor fluctuation onset, years	6.3 ± 3.9	7.3 ± 5.6	5.5 ± 2.6	0.8846 * 0.3851 ^#^
Time from PD diagnosis to morning akinesia, years	7.6 ± 5.7	3.5 ± 1.7	6.8 ± 2.5	0.1486 * 1.0000 ^#^
Time from PD diagnosis to dyskinesia, years	7.9 ± 5.1	6.6 ± 5.3	8.1 ± 3.0	0.3731 * 0.5456 ^#^
Morning akinesia	17 (58.6)	5 (45.5)	28 (84.8)	0.6915 * 0.0369 ^#^
***LCIG initiation***				
Age at LCIG therapy initiation, years	65.3 ± 7.8	67.0 ± 8.9	63.6 ± 8.6	0.5653 * 0.5154 ^#^
Time from PD diagnosis to LCIG initiation, years	10.7 ± 5.2	11.3 ± 6.0	9.5 ± 3.6	0.8723 * 0.5036 ^#^
Reason for LCIG therapy initiation, n (%)				
Disabling motor fluctuations/Off periods Uncontrolled dyskinesia Lack of efficacy of previous treatment Decrease in quality of life	28 (96.6%) 14 (48.3%) 5 (7.2%) 5 (17.2%)	11 (100.0%) 6 (54.5%) 9 (81.8%) 9 (81.8%)	33 (100.0%) 23 (69.7%) 3 (9.1%) 2 (6.1%)	
Dopamine agonists discontinued prior to considering LCIG initiation, n (%)	14 (48.3%)	7 (63.6%)	8 (24.2%)	0.385 * 0.0484 ^#^
***Study visit***				
Age at study visit, years	68.3 ± 7.1	69.5 ± 9.2	65.9 ± 8.4	0.8213 * 0.3549 ^#^
Morning akinesia	10 (34.5)	0 (0.0)	3 (9.1)	0.0387 0.0301
Wearing off, n (%)	22 (75.9%)	10 (90.9%)	32 (97.0%)	0.2881 * 0.0134 ^#^
Dyskinesia, n (%)	19 (65.5%)	8 (72.7%)	25 (75.8%)	0.6638 * 0.3754 ^#^
Co-morbidities at patient visit, n (%)				
Hypertension Cardiovascular disease Depression Cognitive dysfunction Sleep disorders Diabetes mellitus Chronic gastrointestinal disease Orthostatic hypotension Chronic pulmonary disease Fatigue Polyneuropathy/Neuropathy Any malignancy Skin disease	5 (17.2%) 2 (6.9%) 4 (13.8%) 3 (10.3%) 1 (3.4%) 1 (3.4%) 0 (0.0%) 1 (3.4%) 0 (0.0%) 2 (6.9%) 1 (3.4%) 1 (3.4%) 0 (0.0%)	7 (63.6%) 4 (36.4%) 3 (27.3%) 3 (27.3%) 5 (45.5%) 1 (9.1%) 2 (18.2%) 1 (9.1%) 1 (9.1%) 1 (9.1%) 0 (0.0%) 1 (9.1%) 1 (9.1%)	7 (21.2%) 7 (21.2%) 2 (6.1%) 2 (6.1%) 1 (3.0%) 2 (6.1%) 1 (3.0%) 0 (0.0%) 2 (6.1%) 0 (0.0%) 1 (3.0%) 0 (0.0%) 0 (0.0%)	

The table includes only patients with all data available in their records. * *p*-value for comparison of monotherapy vs monotherapy with night medication ^#^ *p*-value for comparison of monotherapy vs add-on therapy. Results are displayed as mean ± standard deviation, if not otherwise specified. LCIG = levodopa-carbidopa intestinal gel; N/n(%) = number (percentage) of patients; PD = Parkinson’s disease.

**Table 2:** Baseline disease characteristics by LCIG treatment duration at patient visit.

Disease Characteristics	≥1 to <2 Years	≥2 to <3 Years	≥3 to <4 Years	≥4 to <5 Years	≥5 Years
Duration of PD at LCIG initiation, mean number of years (SD)	9.2 (4.7) N = 46	9.4 (5.1) N = 20	10.4 (5.8) N = 12	11.1 (8.0) N = 7	9.4 (3.6) N = 10
“Off” time duration at LCIG initiation, mean number of hours/day (SD)	6.7 (3.8) N = 35	6.0 (3.4) N = 10	6.5 (4.3) N = 10	5.0 (0.9) N = 6	4.8 (0.8) N = 5
Duration of dyskinesia at LCIG initiation, mean number of hours/day (SD)	2.0 (2.0) N = 36	3.9 (5.3) N = 11	2.7 (2.3) N = 10	2.2 (1.7) N = 6	3.4 (2.1) N = 5
No or mild dyskinesia at LCIG initiation, N (%)	9 (64.3%) N = 14	2 (66.6%) N = 3	0 N = 2	0 N = 4	1 (12.5%) N = 8
Moderate to severe dyskinesia at LCIG initiation, N (%)	4 (28.6%) N = 14	1 (33.3%) N = 3	2 (100%) N = 2	3 (75%) N = 4	5 (62.5%) N = 8
UPDRS 5 at LCIG initiation—Stage 3 and 4, N (%)	22 (100%) N = 22	8 (88.9%) N = 9	8 (100%) N = 8	4 (100%) N = 4	6 (85.7%) N = 7
UPDRS 5 at LCIG initiation—Stage 5, N (%)	0 N = 22	1 (11.1%) N = 9	0 N = 8	0 N = 4	1 (14.3%) N = 7

LCIG = levodopa-carbidopa intestinal gel; PD = Parkinson’s Disease; UPDRS = Unified Parkinson’s Disease Rating Scale.

**Table 3:** Changes in the “Off” time and “On” time with dyskinesia with LCIG treatment.

	n	%	Mean Change (Hours)	SD	Mean Change (%)	SD
**Change in “Off” time at 12 months from LCIG initiation**						
LCIG monotherapy	13	31.7	−3.2	2.5	−56.3	30.6
LCIG monotherapy + night medication	3	7.3	−8.0	2.6	−80.6	4.8
LCIG + add-on without night medication	19	46.3	−4.0	1.6	−77.1	21.6
LCIG + add-on including night medication	6	14.6	−9.0	4.8	−74.0	23.9
Total population	41	100	−4.8	3.3	−70.3	25.7
**Change in time with dyskinesia at 12 months from LCIG initiation**						
LCIG monotherapy	24	35.8	0.0	3.6	0.9	178.1
LCIG monotherapy + night medication	2	3.0	2.0	0.0	25.0	N/A
LCIG + add-on without night medication	33	49.2	−1.0	1.9	−56.6	36.1
LCIG + add-on including night medication	8	11.9	−1.5	6.9	2.4	110.7
Total population	67	100	−0.6	3.4	−25.2	118.2

LCIG = levodopa/carbidopa intestinal gel.

## Data Availability

The data used to support the findings are available from the corresponding author by reasonable request. Previously reported data (primary manuscript, overall study results) were used to support this study and are available at doi:10.1002/mds.28596. The overall study results are cited in the text as reference #26.

## Supplementary Materials

The following are available online at https://www.mdpi.com/article/10.3390/brainsci11121566/s1, Table S1: Real-world efficacy and safety of switching to bictegravir/emtricitabine/tenofovir alafenamide in older people living with HIV; Table S2: Reasons for start and discontinuation of add-on Parkinson’s disease medication up to 12 months following LCIG initiation; Table S3: UPDRS total and subdomain scores, non-motor symptoms and cognitive mental status at LCIG initiation and at study visit, by study groups; Table S4: Duration of “Off” time and “On” time with dyskinesia at LCIG initiation by treatment groups at 12 months; Table S5: Patient reported outcomes as assessed at study visit, by study groups; Table S6: Occurrence of adverse drug reactions from LCIG therapy initiation up to the study visit.
