# Supplementary material for: Advanced Parkinson’s Disease Treatment Simplification and Long-Term Outcomes with Levodopa Carbidopa Intestinal Gel: COSMOS Romanian Subanalysis
Source: Brain Sci. 2021 Nov 27;11(12):1566. doi: 10.3390/brainsci11121566 (PMC8699449; doi:10.3390/brainsci11121566)
Supplement: Supplementary file 1 [file brainsci-11-01566-s001.zip › 1.pdf]

Supplementary Table S1. Number of patients with add-on PD medication (without rescue medication) at LCIG initiation

Medication	N (%)
Dopamine agonists (any intake)	23 (48.9)
Rotigotine	17 (36.2)
Pramiprexole	4 (8.5)
Ropinoprile	3 (6.4)
Levodopa (any intake)	19 (40.4)
Levodopa/carbidopa	9 (19.1)
Levodopa/benserazide	7 (14.9)
Levodopa/carbidopa/entacapone	5 (10.6)
Rasagiline	19 (40.4)
Amantadine	1 (2.1)

% are calculated from the total number of 47 patients with any intake of add-on PD medications; multiple answers were allowed.

Supplementary Table S2. Reasons for start and discontinuation of add-on Parkinson's disease medication up to 12 months following LCIG initiation

	**LCIG monotherapy at 12 months**<br><br>**N=29**	**LCIG monotherapy with night medication at 12 months**<br><br>**N=11**	**LCIG+add-on medication at 12 months**<br><br>**N=33**
Reasons for start of add-on PD medication			
First 3 months after LCIG initiation, n (%)	1 (3.4%)	8 (72.7%)	6 (18.2%)
Night medication	1 (3.4%)	8 (72.7%)	2 (6.1%)
Rescue medication	0 (0.0%)	3 (27.3%)	1 (3.0%)
To improve a specific symptom	0 (0.0%)	0 (0.0%)	3(9.1%)

Other	0 (0.0%)	0 (0.0%)	2 (6.1%)
3 to 6 months after LCIG initiation, n (%)	0 (0.0%)	0 (0.0%)	2 (6.1%)
Lack of efficacy	0 (0.0%)	0 (0.0%)	1 (3.0%)
Night medication	0 (0.0%)	0 (0.0%)	1 (3.0%)
To improve a specific symptom	0 (0.0%)	0 (0.0%)	1 (3.0%)
6 to 9 months after LCIG initiation, n (%)	0 (0.0%)	1 (9.1%)	0 (0.0%)
Night medication	0 (0.0%)	1 (9.1%)	0 (0.0%)
Reason for discontinuation of add-on PD medication			
First 3 months after LCIG initiation, n (%)	1 (3.4%)	2 (18.2%)	0 (0.0%)
LCIG initiation	1 (3.4%)	2 (18.2%)	0 (0.0%)
Lack of efficacy	0 (0.0%)	1 (9.1%)	0 (0.0%)
Not needed anymore	0 (0.0%)	1 (9.1%)	0 (0.0%)
Intolerability	0 (0.0%)	1 (9.1%)	0 (0.0%)
3 to 6 months after LCIG initiation, n (%)	2 (6.9%)	0 (0.0%)	1 (3.0%)
LCIG initiation	1 (3.4%)	0 (0.0%)	0 (0.0%)
Lack of efficacy	1 (3.4%)	0 (0.0%)	0 (0.0%)
Not needed anymore	0 (0.0%)	0 (0.0%)	1 (3.0%)
6 to 9 months after LCIG initiation, n (%)	0 (0.0%)	0 (0.0%)	1 (3.0%)
LCIG initiation	0 (0.0%)	0 (0.0%)	1 (3.0%)
9 to 12 months after LCIG initiation, n (%)	1 (3.4%)	1 (9.1%)	0 (0.0%)
Lack of efficacy	0 (0.0%)	1 (9.1%)	0 (0.0%)
Not needed anymore	0 (0.0%)	1 (9.1%)	0 (0.0%)
Patient / Caregiver preference	1 (3.4%)	0 (0.0%)	0 (0.0%)

LCIG=levodopa-carbidopa intestinal gel; N/n(%)=number (percentage) of patients; PD=Parkinson's disease; MAO=monoamine oxidase; NMDA= N-methyl-D-aspartate receptor

Supplementary Table S3. UPDRS total and subdomain scores, non-motor symptoms and cognitive mental status at LCIG initiation and at study visit, by study groups

	**LCIG monotherapy at 12 months**			**LCIG monotherapy with night medication at 12 months**			**LCIG+ add-on medication at 12 months**		
	Before LCIG initiation	Study visit	p-value	Before LCIG initiation	Study visit	p-value	Before LCIG initiation	Study visit	p-value
UPDRS total score	50.5±31.6	52.9±25.3	0.2500	127.0	55.5±24.8	1.0000	39.0	38.6±20.1	1.0000
Mood (UPDRS I)	4.5±5.7	2.8±2.8	1.0000	-	2.7±1.7		3.0	2.5±2.0	1.0000
**ADL (UPDRS II)**	16.7±6.7	16.6±7.6	0.2012	5.0	18.4±7.9	1.0000	**16.3±7.2**	**13.1±6.6**	**0.0469**
**Motor symptoms (UPDRS III)**	**15.2±10.0**	**29.7±14.6**	**0.0117**	8.0	28.8±16.3	1.0000	19.1±8.7	20.1±12.2	0.8125
Complications of therapy (UPDRS IV)	5.3±4.9	3.8±3.7	0.9844	8.0	5.5±3.4	1.0000	2.9±1.1	2.9±1.7	0.9063
Modified Hoehn & Yahr Stage (UPDRS V)									
Stage 1	0 (0.0)	0 (0.0%)		1 (25.0)	1 (9.1%)		0 (0.0)	0 (0.0%)	
Stage 1.5	1 (4.8)	1 (3.4%)		0 (0.0)	0 (0.0%)		0 (0.0)	0 (0.0%)	
Stage 3	12 (57.1)	16 (55.2%)		1 (25.0)	3 (27.3%)		8 (72.7)	25 (78.1%)	
Stage 4	7 (33.3)	11 (37.9%)		2 (50.0)	7 (63.6%)		2 (18.2)	7 (21.9%)	
Stage 5	1 (4.8)	1 (3.4%)		0 (0.0)	0 (0.0%)		1 (9.1)	0 (0.0%)	
NMSS total score	NE	34.9±40.6	NE	NE	57.3±39.0	NE	NE	34.1±29.2	NE
**MMSE**	**28.3±3.0**	**27.0±2.9**	**<0.0001**	**26.3±2.2**	**24.7±4.2**	**0.0020**	**28.7±1.9**	**27.5±3.5**	**<0.0001**

*p-value for comparison of values before LCIG initiation and at study visit

Results are displayed as mean±standard deviation, if not otherwise specified.

LCIG=levodopa-carbidopa intestinal gel; MMSE=Mini-Mental State Examination; N=number of patients; NE=not evaluated; NMSS=non-motor symptom scale; UPDRS=Unified Parkinson's Disease Rating Scale

Supplementary Table S4. Duration of "Off" time and "On" time with dyskinesia at LCIG initiation by treatment groups at 12 months

	n	%	Mean number of hours	SD
**Duration of "Off" time at LCIG initiation**				
LCIG monotherapy	23	34.9	5.9	3.5
LCIG monotherapy + night medication	3	4.5	10.0	3.5
LCIG + add-on without night medication	32	48.5	4.9	1.0
LCIG + add-on including night medication	8	12.1	11.6	4.3
Total population	66	100	6.3	3.5
**Duration of dyskinesia at LCIG initiation**				
LCIG monotherapy	24	35.3	2.3	1.9
LCIG monotherapy + night medication	2	2.9	4.0	5.7
LCIG + add-on without night medication	33	48.5	2.1	1.9
LCIG + add-on including night medication	9	13.2	4.2	5.9
Total population	68	100	2.5	2.9

LCIG=levodopa-carbidopa intestinal gel

Supplementary Table S5. Patient reported outcomes as assessed at study visit, by study groups

	**LCIG monotherapy at 12 months N=29**	**LCIG monotherapy with night medication at 12 months N=11**	**LCIG+add-on medication at 12 months N=33**	**p-value**
PDQ-8 summary index score	44.9±21.2	38.9±18.2	39.9±20.9	0.5672*<br>0.3960#
QUIP-RS total score	6.7±10.1	11.8±12.3	7.2±14.1	0.1421*<br>0.5317#

Gambling	0.4±1.3	0.0±0.0	0.5±1.7	
Sex	0.7±1.9	1.3±2.5	1.3±2.6	
Buying	0.8±1.6	3.6±3.6	1.4±3.0	
Eating	1.5±2.5	2.0±2.9	1.4±2.8	
Hobbyism/punding	2.1±4.1	4.1±6.0	2.4±5.5	
Medication use	1.8±3.2	2.4±3.7	1.5±3.9	
Total ICD score	2.9±4.6	6.4±5.9	3.7±7.0	
PDSS-2 total score	21.7±11.7	22.0±7.9	22.6±10.9	0.7249*<br>0.6832
BMQ				
Overuse	2.5±1.1	3.0±1.0	2.4±1.0	0.3348*<br>0.3845#
Harm	2.3±1.0	2.4±1.0	2.0±0.9	0.8555*<br>0.1245#
Necessity	4.6±0.4	4.7±0.5	4.2±0.9	0.4390*<br>0.1077#
Concern	3.0±1.1	2.9±0.8	2.9±1.2	0.9360*<br>0.8063#

*p-value for comparison of monotherapy against monotherapy with night medication

#p-value for comparison of monotherapy against add-on therapy

Results are displayed as mean±standard deviation, if not otherwise specified.

N=number of patients; LCIG=levodopa/carbidopa intestinal gel; PDQ-8=Parkinson's Disease Questionnaire 8-item; QUIP-RS=Questionnaire for Impulsive-Compulsive Disorders in Parkinson's Disease – Rating Scale; PDSS-2=Parkinson's Disease Sleep Scale-2; BMQ=Beliefs Medication Questionnaire

Supplementary Table S6. Occurrence of adverse drug reactions from LCIG therapy initiation up to the study visit

	**LCIG initiation N=95**	**LCIG maintenance treatment N=95**
Any adverse reaction, n (%)	1 (1.1)	3 (3.2)
Polyneuropathy, n (%)	1 (1.1)	1 (1.1)
Stoma site discharge, n (%)	0	1 (1.1)
Embedded device, n (%)	0	1 (1.1)

LCIG=levodopa/carbidopa intestinal gel; N/n (%)=number (percentage) of patients
